# Allele loss and mutation screen at the Peutz-Jeghers (*LKB1*) locus (19p13.3) in sporadic ovarian tumours

**DOI:** 10.1038/sj.bjc.6690323

**Published:** 1999-04

**Authors:** Z-J Wang, M Churchman, I G Campbell, W-H Xu, Z-Y Yan, W G McCluggage, W D Foulkes, I P M Tomlinson

**Affiliations:** Tumour Genetics Group, Nuffield Department of Clinical Medicine, University of Oxford, John Radcliffe Hospital, Oxford OX3 9DU, UK; Department of Surgery, The First School of Medicine, Beijing Medical University, China; Department of Obstetrics and Gynaecology, University of Southampton, Princess Anne Hospital, Coxford Rd, Southampton SO16 5YA, UK; Department of Pathology, Royal Hospitals NHS Trust, Grosvenor Road, Belfast BT12 6BL, UK; Division of Medical Genetics, Departments of Medicine and Human Genetics, McGill University, Montreal General Hospital, Montreal, Quebec, Canada H3G 1A4; Molecular and Population Genetics Laboratory, Imperial Cancer Research Fund, 44 Lincoln's Inn Fields, London WC2A 3PX, UK

**Keywords:** Peutz-Jeghers, *LKB1/STK11*, ovary, adenocarcinoma, granulosa cell

## Abstract

Germline mutations in the *LKB1* (*STK11*) gene (chromosome sub-band 19p13.3) cause characteristic hamartomas and pigmentation to develop in patients with Peutz-Jeghers syndrome. Peutz-Jeghers syndrome carries an overall risk of cancer that may be up to 20 times that of the general population and Peutz-Jeghers patients are at increased risk of benign and malignant ovarian tumours, particularly granulosa cell tumours. Loss of heterozygosity (allele loss, LOH) has been reported in about 50% of ovarian cancers on 19p13.3. *LKB1* is therefore a candidate tumour suppressor gene for sporadic ovarian tumours. We found allele loss at the marker *D19S886* (19p13.3) in 12 of 49 (24%) sporadic ovarian adenocarcinomas. Using SSCP analysis, we screened ten ovarian cancers with LOH, 35 other ovarian cancers and 12 granulosa cell tumours of the ovary for somatic mutations in *LKB1*. No variants were detected in any of the adenocarcinomas. Two mutations were detected in one of the granulosa cell tumours: a mis-sense mutation affecting the putative ‘start’ codon (ATG → ACG, M1T); and a silent change in exon 7 (CTT → CTA, leucine). Like *BRCA1* and *BRCA2*, therefore, it appears that *LKB1* mutations can cause ovarian tumours when present in the germline, but occur rarely in the soma. The allele loss on 19p13.3 in ovarian cancers almost certainly targets a different gene from *LKB1*. © 1999 Cancer Research Campaign


*British Journal of Cancer* (1999) **80**(1/2), 70–72
© 1999 Cancer Research Campaign
Article no. bjoc.1998.0323

### Allele loss and mutation screen at the Peutz-Jeghers (*LKB1*) locus (19p13.3) in sporadic ovarian tumours

**Z-J Wang[1,2], M Churchman[1], IG Campbell[3], W-H Xu[2], Z-Y Yan[2], WG McCluggage[4], WD Foulkes[5] and IPM Tomlinson[1,6]**

[1]Tumour Genetics Group, Nuffield Department of Clinical Medicine, University of Oxford, John Radcliffe Hospital, Oxford OX3 9DU, UK; [2]Department of Surgery, The First School of Medicine, Beijing Medical University, China; [3]Department of Obstetrics and Gynaecology, University of Southampton, Princess Anne Hospital, Coxford Rd, Southampton SO16 5YA, UK; [4]Department of Pathology, Royal Hospitals NHS Trust, Grosvenor Road, Belfast BT12 6BL, UK; [5]Division of Medical Genetics, Departments of Medicine and Human Genetics, McGill University, Montreal General Hospital, Montreal, Quebec, Canada H3G 1A4; [6]Molecular and Population Genetics Laboratory, Imperial Cancer Research Fund, 44 Lincoln's Inn Fields, London WC2A 3PX, UK

**Summary** Germline mutations in the *LKB1* (*STK11*) gene (chromosome sub-band 19p13.3) cause characteristic hamartomas and pigmentation to develop in patients with Peutz-Jeghers syndrome. Peutz-Jeghers syndrome carries an overall risk of cancer that may be up to 20 times that of the general population and Peutz-Jeghers patients are at increased risk of benign and malignant ovarian tumours, particularly granulosa cell tumours. Loss of heterozygosity (allele loss, LOH) has been reported in about 50% of ovarian cancers on 19p13.3. *LKB1* is therefore a candidate tumour suppressor gene for sporadic ovarian tumours. We found allele loss at the marker *D19S886* (19p13.3) in 12 of 49 (24%) sporadic ovarian adenocarcinomas. Using SSCP analysis, we screened ten ovarian cancers with LOH, 35 other ovarian cancers and 12 granulosa cell tumours of the ovary for somatic mutations in *LKB1*. No variants were detected in any of the adenocarcinomas. Two mutations were detected in one of the granulosa cell tumours: a mis-sense mutation affecting the putative 'start' codon (ATG → ACG, M1T); and a silent change in exon 7 (CTT → CTA, leucine). Like *BRCA1* and *BRCA2*, therefore, it appears that *LKB1* mutations can cause ovarian tumours when present in the germline, but occur rarely in the soma. The allele loss on 19p13.3 in ovarian cancers almost certainly targets a different gene from *LKB1*.

**Keywords**: Peutz-Jeghers; *LKB1/STK11*; ovary; adenocarcinoma; granulosa cell

Mendelian diseases which predispose to ovarian tumours include familial breast/ovarian cancer (resulting from germline *BRCA1* and *BRCA2* mutations), Gorlin syndrome (resulting from *PTCH* mutations), hereditary non-polyposis colon cancer (HNPCC, resulting from mismatch repair gene mutations) and Peutz-Jeghers syndrome (PJS, MIM175200). PJS is characterized by hamartomatous polyps of the gastrointestinal tract and other epithelia, and by freckling of the lips, buccal mucosa and other sites (Tomlinson and Houlston, 1997). PJS patients have an increased risk of neoplasia of multiple sites. This risk may approach a 20-fold increase over the general population if all organs are considered, although the increased risk for any particular site is necessarily more modest (Murday and Slack, 1989).

The gene for PJS has recently been shown to be a serine/threonine kinase, known as *LKB1* or *STK11* (Genbank U63333), which maps to chromosome sub-band 19p13.3 (Hemminki et al, 1997, 1998). This gene acts as a tumour suppressor in the hamartomatous polyps of PJS patients and probably also acts as a tumour suppressor in the other neoplasms that develop in PJS patients (Wang et al, 1999). It is not clear whether these neoplasms develop from hamartomas, or whether the *LKB1* locus plays a role in a different genetic pathway of tumour growth, although the former is more likely.

*Received 13 July 1998*
*Revised 12 October 1998*
*Accepted 22 October 1998*

*Correspondence to:* IPM Tomlinson, Molecular and Population Genetics Laboratory, 4th Floor, Imperial Cancer Research Fund, PO Box 123, 44 Lincoln's Inn Fields, London WC2A 3PX, UK

Peutz-Jeghers patients are at increased risk of a number of gynaecological neoplasms. These include benign and malignant ovarian lesions, especially granulosa cell tumours (GCTs), in addition to adenoma malignum of the cervix and endometrial adenocarcinoma. Ovarian adenocarinomas show a relatively high frequency of loss of heterozygosity (allele loss, LOH) on chromosome 19p13.3 (Sato et al, 1991; Osborne and Leech, 1994; Amfo et al, 1995; Pejovic, 1995). *LKB1* is therefore a good candidate for involvement in the pathogenesis of sporadic tumours of the ovary. We have analysed a set of sporadic adenocarcinomas of the ovary for allele loss on chromosome 19p13.3 and then screened these tumours and a set of ovarian granulosa cell tumours for mutations in the *LKB1* gene.

### METHODS

Using standard methods, DNA was extracted from 60 samples of unselected, fresh-frozen sporadic adenocarcinomas of the ovary and matched normal tissue or blood. After microdissection to enrich for tumour material, DNA was extracted from fixed, paraffin-embedded samples of 12 GCTs of the ovary using proteinase K digestion and the Qiagen tissue extraction kit. None of these cases had known clinical or familial features suggestive of PJS. Standard clinicopathological data (patient age, and tumour grade and stage) were available from hospital records.

For allele loss analysis at *LKB1*, the *D19S886* microsatellite marker was used; this maps within 500 kb of *LKB1* (http://www-bio.llnl.gov/). About 50 ng of DNA from paired tumour/normal samples from the ovarian adenocarcinoma patients were amplified using the polymerase chain reaction (PCR) under standard conditions. The forward primer had previously been end-labelled with

$\gamma$-$^{32}$ATP using 3U of T4 polynucleotide kinase. Radio-labelled products were electrophoresed through 6% denaturing polyacrylamide gels, dried and exposed to X-ray film for 24 h. Quantitation of PCR products from tumours and the corresponding constitutional DNA was achieved using the Molecular Dynamics phoshorimager and software. Allele loss was scored if the area under an allelic peak was reduced to < 50% of its original value (relative to the other allele), thus making allowance for the presence of contaminating stromal tissue or inflammatory infiltrate in some of the tumours.

Single-strand conformational polymorphism (SSCP) analysis was performed on the tumour samples as the method of mutation screening at *LKB1*. For the adenocarcinomas, published oligonucleotides and reaction conditions were used for exon-by-exon amplification of *LKB1* in the PCR (Wang et al, 1998). For the GCTs, new oligonucleotides were designed to produce shorter PCR target sequences which were more likely to amplify successfully from fixed archival material in the PCR (Table 1). A PCR protocol of 94°C (3 min) × 1, 94°C (1 min)/Ta °C (1 min)/72°C (1 min) × 35, 72°C (5 min) × 1 was used for the GCTs, with the addition of 0.02% dimethyl sulphoxide (DMSO) and 0.1% bovine serum albumin (BSA) to the reaction where necessary. PCR products were heated to 90°C for 5 min and subjected to electrophoresis on an 8% acrylamide gel (37.5:1 acrylamide: bisacrylamide, 10% glycerol) under non-denaturing conditions at 20 mA for about 16 h. DNA was detected by silver-staining of gels using standard methods. For all tumours with possible mutations according to SSCP analysis, the appropriate exon was reamplified from genomic DNA in the PCR, and purified PCR products were sequenced in forward and reverse orientation using the ABI Ready Reaction Dye Terminator Cycle Sequencing kit and the 373 sequencer. All sequencing reactions were performed in duplicate and alongside samples with wild-type genotypes, and patient samples with known germline mutations in *LKB1*.

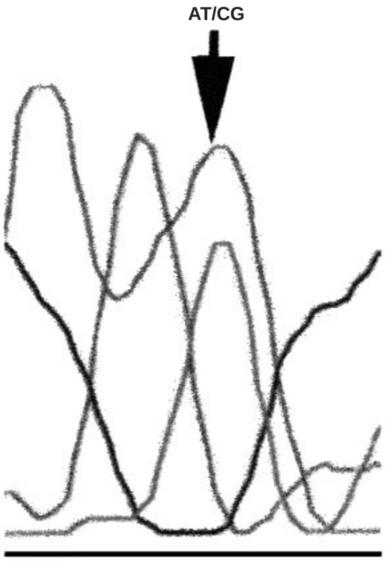

**Figure 1** M1T mutation in granulosa cell humour. Wild type sequence is not shown

### RESULTS AND DISCUSSION

Allele loss was found at *D19S886* in 12 of 49 (24%) informative ovarian adenocarcinomas (out of the total of 60 studied). This is apparently a somewhat lower frequency of allele loss on 19p than found in some studies, but inspection of the data from these other studies shows that our results are in agreement if the previous analysis of multiple markers on 19p, the sample sizes used and the different tumour types studied are taken into account. Sato et al (1991) found allele loss at *D19S20* (4 cM from *D19S886*) in five of 19 (26%) adenocarcinomas of the ovary (map data from

**Table 1** Oligonucleotides used for study of paraffin-embedded archival tumour material

| Exon | Oligo (F,R) | Sequence | Ta | Product length |
|---|---|---|---|---|
| 1 | GB1727 | AGG GCT GGC GGC GGG ACT CC | 58 | 211 |
| | GB1936 | TCC TTC ACC TTG CCG TAA GAG C | | |
| | GB1920 | GAC CTG CTG GGG GAA GGC TCT T | 58 | 170 |
| | GB2090 | AAC CAT CAG CAC CGT GAC TGG | | |
| 2 | GC1289 | CTG ATA CAC CCC TGT CCT CTC TGT C | 54 | 120 |
| | GC1409 | AGG CCC CGC GGT CCC AAC AC | | |
| 3 | GD5531 | CTC CAG AGC CCC TTT TCT G | 59 | 255 |
| | GD5786 | TCA ATG ACT ATC AGG CCA CG | | |
| 4/5 | GA826 | GGC CCC AGG ACG GGT GTG TG | 58 | 160 |
| | GA986 | CCC TAG CAC GTG CCT ACC TC | | |
| | GA951 | GTG GCA CCC TCA AAA TCT CC | 57 | 183 |
| | GA1134 | TCC AGG CCG TTG GCA ATC TC | | |
| | GA1071 | ACC CGT TCG CGG CGG ACG | 58 | 152 |
| | GA1223 | AGT GTG CGT GTG GTG AGT GC | | |
| 6 | GA1659 | TGA CTG ACC ACG CCT TTC TT | 57 | 218 |
| | GA1877 | CCC CCA ACC CTA CAT TTC TG | | |
| 7 | GA2412 | CTC CTC GCC GGC TTC TCC TC | 62 | 155 |
| | GA2567 | CCC CAC CAC GCC CTG CTC TA | | |
| 8 | GA3439 | GAC AGG CGC CAC TGC TTC TG | 60 | 251 |
| | GA3690 | GGA CAT CCT GGC CGA GTC AG | | |
| 9 | GE001 | GTA AGT GCG TCC CCG TGG TG | 59 | 337 |
| | GE338 | GTG GCA TCC AGG CGT TGT CC | | |

*© Cancer Research Campaign 1999*

ftp://cedar.genetics.soton.ac.uk/pub/chrom19/map). Osborne and Leech (1994) found allele loss at *D19S177* (20 cM proximal to *D19S886*) in four of ten (40%) ovarian adenocarcinomas of the ovary. Amfo et al (1995) detected allele loss (in addition to one putative rearrangement) in two of nine (22%) adenocarcinomas at the *INSR* locus (23 cM proximal to *D19S886*). Our results do not differ significantly from any of these other studies ($\chi^2$ test, details not shown). *LKB1* therefore remained an excellent candidate gene for acting as a tumour suppressor in ovarian tumorigenesis.

In a set of ten adenocarcinomas with LOH, in 35 other adenocarcinomas selected at random, and in 12 GCTs, a small number of variant bands suggestive of somatic mutations at *LKB1* was detected on SSCP analysis. In all the carcinomas, these putative bandshifts were shown on sequencing to be intronic polymorphisms or other intronic variants with no predicted effect on mRNA or protein. A commonly observed biallelic polymorphism (C/G, heterozygosity 44% in a sample of 34 individuals) was found in intron 7 at the +8 splice donor site. This polymorphism is found at approximately equal allele frequencies (details not shown) in normal individuals, in PJS patients and in tumours, and it almost certainly has no effect on mRNA splicing; it may, however, be useful for future allele loss studies at the *LKB1* locus. No *LKB1* mutations were found in the adenocarcinomas. Two mutations were, however, detected in one of the granulosa cell tumours (Figure 1): a mis-sense mutation affecting the putative 'start' codon (ATG → ACG, M1T); and a silent change in exon 7 (CTT → CTA, leucine). These variants were not present in the germline. Analysis of the sequence showed that this tumour did not exhibit allele loss at *LKB1*. No particular clinicopathological features distinguished this granulosa cell tumour from any of the others.

It is not clear whether or not the M1T mutation in one of the granulosa cell tumours has any pathogenic effect. Certainly, it occurs outside the kinase core (codons 50–337) in which most germline mutations have been found (Hemminki et al, 1998). Although codon 1 provides the best candidate 'start' codon for *LKB1* translation, there are several alternative 'start' codons just downstream (at codons 11, 18 and 22) which may allow near-normal function of the LKB1 protein. Codon 22, in particular, is flanked by sequences which suggest that it could function efficiently as an alternative initiator of translation. It is even possible that the methionine at codon 1 is not the usual 'start' site for *LKB1*. The M1T mutation has not, however, been observed as a variant in over 50 other PJS patients, tumours and normal individuals sequenced for exon 1 of *LKB1*.

SSCP only detects about 80% of mutations (Sheffield et al, 1993; Ravnik et al, 1994; Vidal and Moller, 1994), and this figure may be somewhat lower for some types of point mutation. We have not excluded further possibilities for the involvement of *LKB1* in ovarian tumorigenesis, such as gene silencing by promoter methylation, or hemi- or homozygous deletion of either locus (whether the entire gene or whole exons). Thus, it remains possible that mutations or transcriptional inactivation at *LKB1* occur in a larger proportion of ovarian tumours than we have reported. It remains most likely, however, that – similar to that with the *BRCA1* and *BRCA2* genes in familial breast/ovarian cancer – inherited variation in *LKB1* predisposes to ovarian tumours in PJS, but somatic mutations in the same gene are rarely important for the pathogenesis of sporadic tumours of this site. Occasional *LKB1* mutations with pathogenic effects have been found in cancers of the colon, testis, lung and skin (Dong et al, 1998; Wanger et al, 1998; Rowan et al, 1999; Wang et al, 1999; Ylikorkala et al, 1999).

Although somatic *LKB1* mutations may be important in the pathogenesis of a minority of sporadic granulosa cell tumours, it is most likely that *LKB1* is not mutated in ovarian adenocarcinomas and that the allele loss observed on chromosome 19p13.3 in these cancers targets a different locus from *LKB1*. Whether or not *INSR* is actually the gene involved, the combined data from previous work and our study suggest that allele loss on 19p13.3 in ovarian cancer targets a different locus from *LKB1*. Candidate genes in this region of chromosome 19p include basigin, *CDC34*, *PTPRS*, *AMH*, *ICAM1*, *ICAM3* and *CDKN2D*.

### ACKNOWLEDGEMENTS

We are grateful to the following bodies for support: Imperial Cancer Research Fund (IT, Z-JW); Jane Ashley Trust (IT); Cancer Research Campaign MC; Henry Lester Trust (Z-JW); China Scolarship Council (Z-JW); MGH 175th Anniversary Scholarship (WF).

### REFERENCES

Amfo K, Neyns B, Teugels E, Lissens W, Bourgain C, De Sutter P, Vandamme B, Vamos E and De Greve J (1995) Frequent deletion of chromosome 19 and a rare rearrangement of 19p13.3 involving the insulin receptor gene in human ovarian cancer. *Oncogene* **11**: 351–358

Dong SM, Kim KM, Kim SY, Shin MS, Na EY, Lee SH, Park WS, Yoo NJ, Jang JJ, Yoon CY, Kim JW, Kim SY, Yang YM, Kim SH, Kim CS and Lee JY (1998) Frequent somatic mutations in serine/threonine kinase 11/Peutz-Jeghers syndrome gene in left-sided colon cancer. *Cancer Res* **58**: 3787–3790

Hemminki A, Tomlinson I, Markie D, Jarvinen H, Sistonen P, Bjorkqvist AM, Knuutila S, Salovaara R, Bodmer W, Shibata D, de la Chapelle A and Aaltonen LA (1997) Localization of a susceptibility locus for Peutz-Jeghers syndrome to 19p using comparative genomic hybridization and targeted linkage analysis. *Nat Genet* **15**: 87–90

Hemminki A, Markie D, Tomlinson IPM, Avizienyte E, Roth S, Loukola A, Bignell G, Warren W, Järvinen H, Aminoff M, Höglund P, Pelin K, Ridanpää M, Salovaara R, Olschwang S, Bodmer WF, Olsen A, Stratton MR, de la Chapelle A and Aaltonen LA (1998) A serine/threonine kinase gene defective in Peutz-Jeghers syndrome. *Nature* **391**: 184–187

Murday V and Slack J (1989) Inherited disorders associated with colorectal cancer. *Cancer Surv* **8**: 139–157

Osborne RJ and Leech V (1994) Polymerase chain reaction allelotyping of human ovarian cancer. *Br J Cancer* **69**: 429–438

Pejovic T (1995) Genetic changes in ovarian cancer. *Ann Med* **27**: 73–78

Ravnik GM, Glavac D and Dean M (1994) Sensitivity of single-strand conformation polymorphism and heteroduplex method for mutation detection in the cystic fibrosis gene. *Hum Mol Genet* **3**: 801–807

Rowan A, Bataille V, MacKie R, Healy E, Bicknell D, Bodmer WF and Tomlinson IPM (1999) Somatic mutations in the Peutz-Jeghers (*LKB1*/*STK11*) gene in sporadic malignant melanomas. *J Invest Dermatol* (in press)

Sato T, Saito H, Morita R, Koi S, Lee JH and Nakamura Y (1991) Allelotype of human ovarian cancer. *Cancer Res* **51**: 5118–5122

Sheffield VC, Beck JS, Kwitek AE, Sandstrom DW and Stone EM (1993) The sensitivity of single-strand conformation polymorphism analysis for the detection of single base substitutions. *Genomics* **16**: 325–332

Tomlinson I and Houlston R (1997) Peutz-Jeghers syndrome. *J Med Genet* **34**: 1007–1011

Vidal PA and Moller DE (1994) Comparative sensitivity of alternative single-strand conformation polymorphism (SSCP) methods. *Biotechniques* **17**: 490–492

Wang Z-J, Taylor F, Churchman M, Norbury CG and Tomlinson IPM (1998) Genetic pathways of colorectal carcinogenesis rarely involve the PTEN and LKBI genes outside the inherited hamartoma syndromes. *Am J Pathol* **153**: 363–366

Wang Z-J, Ellis I, Zauber P, Iwama T, Marchese C, Talbot IC, Xue W-H, Yan Z-Y and Tomlinson IPM (1999) Allelic imbalance at the *LKB1* (*STK11*) locus on 19p13.3 in hamartomas, adenomas and carcinomas from patients with Peutz-Jeghers syndrome provides evidence for a hamartoma-(adenoma)-carcinoma sequence. *J Pathol* (in press)

Ylikorkala A, Avizienyte E, Tomlinson IPM, Tiainen M, Roth S, Loukola A, Hemminki A, Johansson M, Sistonen P, Markie D, Neale K, Phillips RKS, Zauber P, Iwama T, Sampson J, Järvinen H, Mäkelä TP, Aaltonen LA (1999) Mutations and impaired function of LKB1 in familial and non-familial Peutz-Jeghers syndrome and a sporadic testicular cancer. *Hum Mol Genet* **8**: 45–51

© Cancer Research Campaign 1999
